# Comparison of quadriceps and hamstring muscle size and strength between young athletes following knee surgery and healthy controls

**DOI:** 10.7717/peerj.20330

**Published:** 2025-11-13

**Authors:** Christopher J. Cleary, Isaiah G. Roepe, Christopher D. Bernard, Traci Smiley, John K. Veazey, Kyle A. Martin, Megan Bechtold, Bryan G. Vopat, Ashley A. Herda

**Affiliations:** 1 Department of Health and Human Physiological Sciences, Skidmore College, Saratoga Springs, NY, United States of America; 2 Department of Orthopedic Surgery and Sports Medicine, University of Kansas Medical Center, Kansas City, KS, United States of America; 3 University of Kansas Health Systems, Overland Park, KS, United States of America; 4 Department of Health, Sport, and Exercise Sciences, University of Kansas Edwards Campus, Overland Park, KS, United States of America

**Keywords:** Rehabilitation, Sports medicine, Injury, Surgery, Ultrasound

## Abstract

**Objectives:**

This study compared the size and strength of the quadriceps and hamstring muscles in young athletes who had undergone previous knee surgery (POST) to sex- and age- matched, healthy controls (SAM).

**Methods:**

A total of 18 (nine POST and nine SAM) participants volunteered to participate in the study’s procedures. Of the nine POST participants, six had underwent anterior cruciate ligament reconstruction, two underwent medial patellofemoral ligament reconstruction and one had undergone patellar tendon repair. Maximal voluntary isometric contractions assessed absolute strength (MVIC_ABS_) of the quadriceps and hamstrings. Muscle size was quantified as muscle cross-sectional area (mCSA) from panoramic ultrasound images. Relative strength (MVIC_REL_) was calculated as a ratio of strength to muscle size. Separate 2-way mixed-factorial analyses of variance leg (operative or non-dominant (O-ND) *vs.* non-operative or dominant (NO-D)) and group (POST *vs.* SAM) assessed statistical differences at *p* ≤ 0.05.

**Results:**

There were no significant two-way interactions (*p*-range: 0.142–0.74) for any variables. Further, there were no significant main effects for the quadriceps (*p*-range: 0.127–0.605) nor was there a main effect for leg in any hamstrings variables (*p*-range: 0.126–0.367). However, the POST group had greater MVIC_ABS_ and MVIC_REL_ than SAM for the hamstrings by 69.8 ± 30.7 N (*p* = 0.037) and 2.21 ± 1.02 N cm^−2^ (*p* = 0.045).

**Conclusions:**

These findings indicate that there was no difference in quadriceps muscle strength or size between the POST and SAM groups. However, the POST group had greater hamstrings strength than SAM yet no differences in muscle size. These results suggest that the rehabilitation program may have been effective in restoring quadriceps function and enhancing hamstrings strength in young athletes following knee surgery. However, future studies should continue to elucidate the physiological effects of knee surgeries in larger, more diverse samples to attenuate the negative musculoskeletal outcomes experienced even after successful surgery and rehabilitation. Yet, these results can be considered as preliminary findings that demonstrate the feasibility of the inclusion of ultrasound imaging in return-to-sport evaluation in a small sample.

## Introduction

Although exact numbers are not known, approximately 10–25% of all youth athletes will suffer from a knee injury at some point during their athletic careers (Louw, Manilall & Grimmer, 2008). Some of these injuries may include, but are not limited to, anterior cruciate ligament injuries, medial patellofemoral ligament ligaments injuries, and patellar tendon injuries (Clifton et al., 2017; Gianotti et al., 2009; Ingram et al., 2008). Following an injury, it is commonly accepted that the athlete will undergo surgery and a period of rehabilitation to resume pre-injury levels of activity.

It is widely accepted that rehabilitation after orthopedic surgery is just as critical as the surgical technique applied, especially with regards to athletes and return to sport. Evidence supporting this is often reported in anterior cruciate ligament (ACL) and other lower extremity (hip, knee, and ankle) injuries. Post-operative rehabilitation following orthopedic knee surgery typically addresses knee function, with phased goals of improving range of motion, muscular strength and size, and sport-specific training (Badawy et al., 2022; Greenberg et al., 2018; Van Melick et al., 2016). Recent systematic reviews demonstrated that patients who underwent ACL reconstruction (ACLR), specifically, had decreased muscle strength and changes in their knee kinematics and function (Badawy et al., 2022; Hart et al., 2016). Specifically, quadriceps muscle isometric strength of the surgical may be reduced by up to 20% at six months after ACL injury when compared to the nonsurgical limb (Lepley, 2015). While the hamstrings muscles can be reduced by up to 30% at one-year post surgery (Oberländer et al., 2013).

From the clinician’s perspective, it is important that the athlete be able to return to a competitive level of sport but in a safe and timely manner. Rehabilitation often culminates in a functional test to determine physical readiness to return to sport (Greenberg et al., 2018; Van Melick et al., 2016). This assessment addresses imbalances between limbs, using limb symmetry index (LSI) as a determinant of readiness (Wellsandt, Failla & Snyder-Mackler, 2017). LSI is “sufficient” when the operative limb is at the strength or function of at least 90% compared to the intact limb. What this does not account for is any detraining that may occur in the intact limb due to the lengthy recovery of the operative limb. Furthermore, the pre-operative performance of the injured limb is often unknown, leading to strength and performance deficits that cannot be quantified.

The literature indicates that asymmetry in quadriceps strength contributes to poor outcomes after knee surgery and increased ACL re-rupture rates (Grindem et al., 2016; Ithurburn et al., 2015; Kyritsis et al., 2016). Poor hamstring strength and function is also associated with poor outcomes and can increase shear forces across the ACL and knee joint (Buckthorpe, 2019; Buckthorpe, 2021). Further, muscle size has been hypothesized to be related to strength in the lower-limbs after ACLR (Kuenze, Blemker & Hart, 2016; Thomas et al., 2016). Therefore, muscle size should be included as a key performance outcome after ACLR. Specifically, accurate measures of muscle size beyond thigh circumference should be included, such as ultrasound-derived cross-sectional area. At present, there are no quantitative studies that evaluate the size and strength of both quadriceps and hamstring muscle mass in the operative leg compared to the contralateral leg or to sex and age-matched healthy controls. Therefore, the purpose of this study was to compare the size and strength of the quadriceps and hamstring muscles in young athletes who had undergone previous knee surgery to matched, healthy controls. It is hypothesized that patients who are “cleared” to return to sport, that have passed the LSI requirements, will be comparable to their matched peers, providing assurance they are truly ready to proceed with pre-injury sport participation.

## Materials & Methods

### Study design

This study was an observational case-control design in which individuals were assessed for quadriceps and hamstrings skeletal muscle size and strength in a single visit. Upon arrival to the facility, height and body mass were recorded with a platform stadiometer (InBody BSM 270B; InBody USA, Cerritos, CA, USA) and digital scale (InBody770; InBody USA, Cerritos, CA, USA). Musculoskeletal ultrasound was used to capture images of the quadriceps and hamstrings on both legs. Subsequently, skeletal muscle strength was measured with a handheld dynamometer (microFET 2; Hoggan Scientific LLC., Salt Lake City, UT, USA) typically used in clinical practice. Leg dominance was determined for all participants with the non-operative leg considered dominant (NO-D) for POST participants and the leg used to kick a ball for the SAM participants and injured/operative leg for POST and contralateral leg for SAM considered nondominant (O-ND). All study procedures were approved by the University of Kansas Medical Center Human Research Protection Program (IRB#: STUDY00148879) and conducted in accordance with the Declaration of Helsinki. All participants provided written informed consent, or assent if the participant was under 18 years old along with parental/guardian consent, and received a copy of their signed consent form prior to participation.

### Participants

Eighteen young adults were recruited to participate in this case control-matched investigation. A total of nine patients were recruited from a local physical therapy clinic after being identified by their practitioner as “cleared”, on average 5.8 ± 1.0 months post-surgery, to return to sport following orthopedic knee surgery rehabilitation (POST), and nine sex- and age-matched participants were subsequently recruited from the local community as healthy controls (SAM). All participants were recruited *via* convenience sampling. The training history of the SAM group was collected, but not reported, which may limit the group comparisons of the present study’s findings. Similarly, due to our sampling methods and the inclusion of only POST athletes that were deemed clear to return to sport may have introduced selection bias. Specific descriptive characteristics of all recruited participants are provided in the Results section below. All participants met the inclusion criteria of 15–40 years of age, body mass index between 18–30 kg/m^2^, and no current ailments or limitations that impacted their ability to complete the study’s assessments. Participants were excluded if they were a smoker, pregnant, had undergone surgery on both legs, and for the SAM participants, if they had any lower-body injury within the past 6 months. An *a priori* power analysis conducted in G*Power (v3.1.9.6; G*Power, Kiel, Germany) determined that a total of 16 participants were required to reach 80% power at an alpha level of 0.05 with a moderate effect size reported from previous research on the difference in quadriceps strength of the involved limb between healthy individuals and post-surgical individuals (Lisee et al., 2019).

### Musculoskeletal ultrasound image acquisition

Individual muscle images were captured by an experienced investigator using a NextGen Logiq e R7 Ultrasound (GE Healthcare, Wauwatosa, WI, USA) with a 4.5 cm wide linear array transducer (Model L4-12t-RS, 3.0−4.2 MHz; GE Healthcare, Wauwatosa, WI, USA). Image scan depth was set to six cm with the ultrasound gain at 68 dB and transducer frequency was 10 MHz for all participants. All images were acquired with the transducer oriented transversely across the muscle belly in panoramic mode to capture mCSA at each site. Prior to image acquisition, water-soluble ultrasound gel (Aquasonic 100; Parker Laboratories Inc., Fairfield, NJ, USA) was applied to the transducer and the participants’ skin to maximize acoustic image clarity. All images were acquired bilaterally for each participant.

For quadriceps image acquisition, the participant laid supine with a foam pad placed under the knees. The vastus lateralis was captured at 50% of the distance between the greater trochanter and lateral epicondyle of the femur (Ahtiainen et al., 2010). The rectus femoris was imaged at 50% of the distance between the greater trochanter and the superior pole of the patella (Ahtiainen et al., 2010). The vastus medialis was measured at 10 cm above the superior pole of the patella, medially (Garcia et al., 2020). For hamstrings image acquisition, the participant was instructed to lay prone on the examination table, where the biceps femoris, semimembranosus, and semitendinosus were captured in a single panoramic image at 50% of the distance between the ischial tuberosity and popliteal crease (Magnusson et al., 1997).

### Ultrasound image analysis

Ultrasound images were analyzed offline using ImageJ (NIH, Bethesda, MD, USA). All images were first calibrated from pixels to cm using a known distance provided on the panoramic image. mCSA (cm^2^) was determined by outlining the entirety of the muscle belly’s border with care taken to ensure no fascia was included in the region of interest. Quadriceps mCSA was determined as the sum of the rectus femoris, vastus medialis, and vastus lateralis mCSA per leg. Similarly, hamstrings mCSA was determined as the sum of the biceps femoris, semimembranosus, and semitendinosus separately per leg. All images were analyzed once by a single experienced image analyst with documented high intra-rater reliability indicated by intraclass correlation coefficients for repeated mCSA analyses of 0.97−0.99 along with high inter-rater reliability of 0.98−0.99 (Cleary et al., 2022).

### Muscle strength

**Figure 1:**
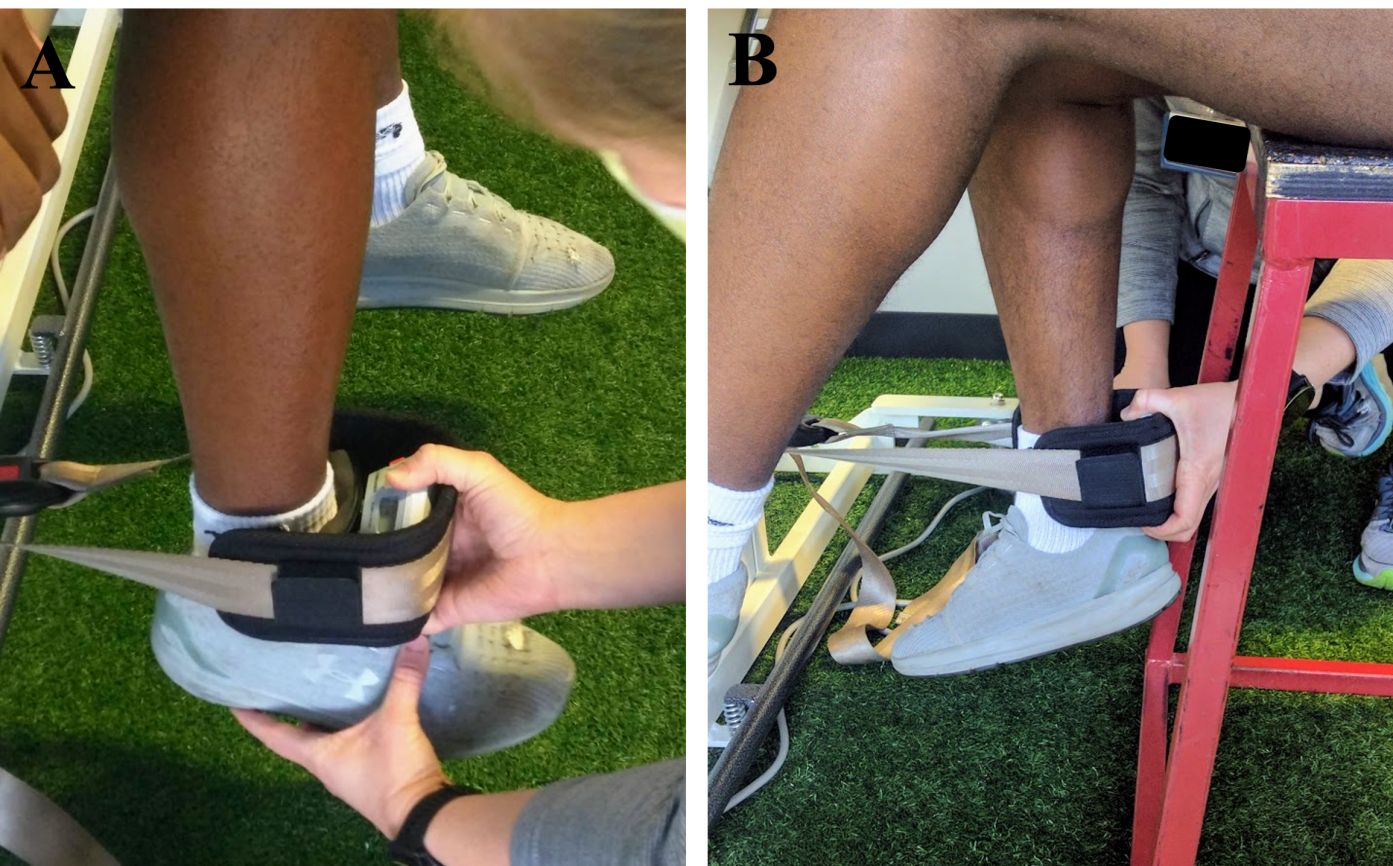
Gait belt secured to examination table for isometric quadriceps (A) and hamstrings (B) strength measurements using a handheld dynamometer.

Skeletal muscle strength of the quadriceps and hamstrings was determined as the peak force (newtons (N)) of maximal isometric voluntary contractions (MVIC). Peak force was recorded from a hand-held dynamometer (microFET 2; Hoggan Scientific LLC., Salt Lake City, UT, USA) secured to the examination table leg using an adjustable gait belt (Fig. 1). This method is the standard protocol for the clinical facility from which patients were recruited and not an isokinetic dynamometer, which is not pragmatic in the facility, yet considered a gold standard for comparison. The participants were seated upright for all test trials with knee and hip angles at ∼90° flexion. Participants were provided approximately 30-seconds of rest between trials and legs. MVIC data were recorded from three repetitions for each action and leg, with the NO-D leg tested first, with the highest force (N) of each being used in subsequent analyses, expressed as absolute strength (MVIC_ABS_), and separately, as relative strength (MVIC_REL_) as force in N produced per unit of muscle from the sum of mCSA (N cm^−2^) for each muscle action.

### Statistical analyses

Six separate 2 × 2 (leg (O-ND *vs.* NO-D) × group (POST *vs.* SAM)) mixed-factorial analyses of variance (ANOVAs) were conducted for quadriceps and hamstrings mCSA, MVIC_ABS_, and MVIC_REL_. In the case of a significant two way interaction, significant main effects were followed up with independent or paired t-tests where appropriate. Effect sizes were generated as partial eta squared (*η*_p_^2^) for the ANOVA interaction and main effect terms, interpreted as trivial (<0.01), small (0.01–0.06), moderate (0.06–0.14) or large (>0.14) (Lakens, 2013), and Cohen’s *d* for t-tests, interpreted as small (<0.2), medium (0.5), or large (>0.8) (Cohen, 1992). All analyses were conducted in R Statistical Software (Version 4.3.0; R Core Team, 2023) in the RStudio Integrated Development Environment (Posit Software, PBC, Boston, MA, USA). Data were considered significant at *p* ≤ 0.05. All raw data and relevant analysis code are available in the Supplemental Files.

## Results

### Participants

Overall, 18 participants were recruited and completed all testing procedures, therefore there were no missing data for any variable. A total of nine individuals that had completed post-orthopedic knee surgery rehabilitation (POST: age = 19.3 ± 6.7 years; height = 177.6 ± 6.9 cm; body mass = 88.02 ± 14.5 kg; two females) and nine sex- and age-matched individuals without a previous orthopedic knee surgery (SAM: age = 19.1 ± 4.8 years; height = 173.5 ± 6.2 cm; body mass = 76.3 ± 13.2 kg; two females) volunteered to participate. All POST participants were cleared to participate in athletic activities by their orthopedic surgeon and clinical rehabilitation team. Six of the POST participants underwent ACLR, two underwent medial patellofemoral ligament (MPFL) reconstruction, and one underwent patellar tendon repair. The POST participants were on average 5.8 ± 1.0 months (range: 4.4 to 7.1 months) post-surgery. The data of all POST participants was collected during their return to sport testing procedures conducted by the patient’s physical therapist.

### Muscle size

There were no significant leg × group interactions (Table 1) for quadriceps (*p* = 0.300) or hamstrings mCSA (*p* = 0.946). Further, there we also no significant main effects for leg (quadriceps: *p* = 0.361; hamstrings: *p* = 0.367) or group (quadriceps: *p* = 0.605; hamstrings: *p* = 0.974) for mCSA of either muscle group.

**Table 1:** Quadriceps and hamstring muscle size and strength and ANOVA results for each variable.

	**POST (*n* = 9)**		**SAM (*n* = 9)**		**Leg × Group**				**Leg** **(O-ND *vs.* NO-D)**			**Group** **(POST *vs.* SAM)**		
	*O-ND*	*NO-D*	*O-ND*	*NO-D*	*F*_(1,16)_	*p*	*η*_p_^2^	*F* (1, 16)		*p*	*d*	*F* (1, 16)	*p*	*d*
**Quadriceps mCSA (cm** ^**2**^ **)**	58.31 ± 7.52	62.62 ± 11.93	63.93 ± 18.04	63.65 ± 16.55	1.146	0.300	0.067	0.885		0.361	0.314	0.279	0.605	0.176
**Hamstrings mCSA (cm** ^**2**^ **)**	30.79 ± 7.22	31.56 ± 7.08	30.62 ± 6.65	31.51 ± 8.16	0.005	0.946	<0.001	0.862		0.367	0.310	0.001	0.974	0.011
**Quadriceps MVIC**_**ABS**_ ** (N)**	374.79 ± 92.76	439.24 ± 157.09	515.75 ± 178.11	510.02 ± 205.01	2.379	0.143	0.129	1.665		0.215	0.430	1.946	0.182	0.465
**Hamstrings MVIC**_**ABS**_ ** (N)**	295.66 ± 88.65	298.87 ± 80.79	214.26 ± 47.64	240.65 ± 42.82	1.598	0.224	0.091	2.616		0.126	0.538	**5.167**	**0.037**	**0.758**
**Quadriceps MVIC**_**REL**_ ** (N cm** ^−2^ **)**	6.46 ± 1.40	7.08 ± 2.45	8.12 ± 1.96	8.18 ± 3.50	0.613	0.445	0.037	0.868		0.365	0.311	1.586	0.226	0.420
**Hamstrings MVIC**_**REL**_ ** (N cm** ^−2^ **)**	9.84 ± 2.94	9.66 ± 2.59	7.19 ± 1.64	7.88 ± 1.40	2.591	0.127	0.139	0.902		0.356	0.316	**4.711**	**0.045**	**0.723**

**Notes.**

Values are presented as mean ± SD.

mCSA muscle cross-sectional area MVIC maximal voluntary isometric contraction POST post-rehabilitative participants SAM sex- and age-matched participants O-ND operative leg for the POST group or non-dominant leg for the SAM group NO-D non-operative leg for the POST group or dominant leg for the SAM group

Bold indicates significant at *p* ≤ 0.05.

### Muscular strength

Similar to the muscle size findings, there were no significant leg ×group interactions (Table 1) for MVIC_ABS_ (quadriceps: *p* = 0.143; hamstrings: *p* = 0.224) or MVIC_REL_ (quadriceps: *p* = 0.445; hamstrings: *p* = 0.127). There were no main effects for leg in either muscle group or muscular strength variable (Table 1). The quadriceps also had no main effect for group in either MVIC_ABS_ or MVIC_REL_. However, for the hamstrings, there was a main effect for group in that the POST group had greater MVIC_ABS_ by 69.8 ± 30.71 N (*p* = 0.037, *d* = 0.758) and MVIC_REL_ by 2.21 ± 1.02 N cm^−2^ (*p* = 0.045, *d* = 0.723) than SAM.

## Discussion

The purpose of this study was to compare the size and strength of the quadriceps and hamstring muscles in young athletes who had undergone previous knee surgery to matched healthy controls. The unique aspect of this study is the simultaneous assessment of muscle size and relative strength using clinically available techniques in young athletes at the time of return to sport testing. The present data indicated there were no differences between legs nor groups for the variables assessed on the quadriceps. However, for the hamstrings, although there were no leg differences for any variable and no group differences in muscle size, MVIC_ABS_ and MVIC_REL_ were 30.8% and 29.3% greater, respectively, in POST than SAM. Overall, these findings suggest that the POST sample of the present study had achieved comparable quadriceps muscle function to a similar and matched healthy control group yet exceeded that healthy control group for hamstrings function.

After knee surgeries, practitioners are primarily interested in limiting between-limb deficits in muscular strength and size (Lepley et al., 2020; Oiestad et al., 2010; Palmieri-Smith, Thomas & Wojtys, 2008). Further, practitioners may be interested in the comparison of the aforementioned variables of post-surgical patients compared to matched healthy control participants without a previous knee surgery (Brown et al., 2021; Sherman et al., 2021). For both comparisons, there is concern that the operative limb will be weaker than the non-operative limb within post-surgical groups and that the post-surgical group will be weaker than healthy control participants. The present study’s findings indicated no differences between limbs or groups of muscle size, strength, and strength expressed relative to muscle size for the quadriceps. Further, there were no leg differences for the hamstrings nor a difference between groups for hamstrings muscle size.

Potentially, this lack of between-limb differences in strength, size, and relative strength may be explained by the timing of testing for the POST participants. As all data were collected during the return to sport testing protocol for the POST participants at an average of ∼5.8-months post-surgery, it is likely that the rehabilitation protocol the POST participants underwent was sufficient to achieve symmetry between the operative and non-operative legs for muscle size, strength, and relative strength. At six months post-surgery, the average asymmetry in quadriceps strength after knee surgeries, specifically ACLR, has been reported to be approximately 23% in a review by Lepley (2015). While for muscle size, Garcia et al. (2020) reported a between-limb difference in mCSA of the rectus femoris and vastus lateralis of 4.9% and 10.3%, respectively, in participants post-ACLR, although there were no differences compared to a healthy control group. Further, for hamstrings muscle size, semitendinosus mCSA at 50% of thigh length, similar to the present study’s methodologies, was smaller than the non-operative limb by 47% and smaller than a similar healthy control group by approximately 50% in patients who were 12–36 months post-ACLR (Morris et al., 2021). As the POST participants of the present study were assessed at the time of their return to sport testing, it is likely that their rehabilitation protocol was structured and sufficient enough to minimize between-limb asymmetries in muscle size and strength, explaining the contrasting results of the present results to those of previous literature. However, it is vital to note that these findings cannot confirm causality of the rehabilitation program’s ability to increase muscular strength.

Further, for muscle strength, the present study’s findings are also in contrast to a recent study that utilized handheld dynamometry and demonstrated a 7.4% difference between legs for quadriceps strength and a 14.5% difference for hamstrings strength at 6-months post-surgery (Welling et al., 2025). Although in Welling et al. (2025) hamstring strength was assessed with the participant in a prone position unlike the seated position in the present study, potentially explaining the discrepancy observed between studies. When compared to similar healthy controls, Holsgaard-Larsen et al. (2014) reported that ACLR patients had a reduced LSI for hamstrings MVIC by approximately 18%. While in the present study, the POST group had greater hamstrings MVIC_ABS_ and MVIC_REL_ than the SAM group. This group difference could be explained by a heightened emphasis on hamstrings strength during rehabilitation in the POST group.

Furthermore, the lack of group and leg differences in quadriceps muscle size and strength can represent a successful rehabilitation protocol for the POST group. The achievement of “symmetrical” quadriceps strength (LSI >90%) is required to be classified as ready to return to sport by the clinic utilized in the present study. Therefore, with consideration of the time period (∼6 months post-surgery) that the POST participants were assessed in the present study, it is not surprising that these participants did not have any differences in quadriceps strength or size compared to the SAM group. Physiologically, the greater hamstrings strength (both relative and absolute) of the POST group compared to SAM likely is explained by the emphasis on total lower limb strength throughout knee surgery rehabilitation (Filbay & Grindem, 2019). Lastly, although training statuses/history of the SAM group were not reported, it could be possible that this sample did not perform much posterior strength training movements, particularly when assessing the values of hamstrings and quadriceps MVIC_ABS_ for each group. Clinically, the present study implies that athlete rehabilitation protocols should consist of basic strength training protocols that emphasize the importance of leg symmetry after knee injuries.

A limitation of the present study is the small sample size potentially limiting statistical power and could explain the lack of statistically significant findings. With such a small sample size, it is potential that the risk of Type II error has been increased. Further, due to the small sample size, the generalizability of our results may be limited. Also, it is possible that selection bias may have occurred as some of the POST athletes who were recruited may have been more motivated throughout their rehabilitation to return to sport. Further, the only functional assessment was maximal muscle strength without adjunctive measurements such as rate of force development and neuromuscular assessments (Angelozzi et al., 2012; Bryant, Kelly & Hohmann, 2008). Lastly, other functional measurements such as single leg jump and hop performance, muscular endurance measurements, or balance and kinematic measurements, were not investigated in the present study. Future studies should consider the addition of other functional measurements, such as balance, agility, and/or jump performance, along with strength and muscle size in larger samples with different surgical types and longer follow-up periods to best investigate the unfortunate musculoskeletal consequences experienced after knee surgeries. Future studies should also assess the training history of post-surgical and healthy participants to help provide context to any potential group differences.

## Conclusions

In conclusion, at the time of return to sport testing in the current sample, quadriceps or hamstrings muscle size, strength, and relative strength were similar between the legs within the post-operative group and to a healthy control group in a small sample. It is important to note that the small sample size of the present study should be interpreted as preliminary findings of musculoskeletal ultrasound’s utility in return to sport evaluations and requires further replication in larger, more diverse cohorts. Overall, these findings suggest that the rehabilitation protocols utilized for the current participants may have been sufficient in restoring symmetrical function after knee surgeries such as ACLR and MPFL. Practically, however, practitioners should continue to stress the importance of adequate rehabilitation training protocols to ensure symmetrical muscular strength after orthopedic knee surgeries. Lastly, to best understand the effects of knee surgeries on musculoskeletal outcomes, future studies should consist of larger samples, with longer follow-up periods after surgery involving different surgical types and include other functional measurements such as rate of force development, balance, and jump testing.

##  Supplemental Information

10.7717/peerj.20330/supp-1 Supplemental Information 1 Raw data

10.7717/peerj.20330/supp-2 Supplemental Information 2 R analysis code for the associated raw data

10.7717/peerj.20330/supp-3 Supplemental Information 3 STROBE

## Additional Information and Declarations

### Data Availability

The following information was supplied regarding data availability: The raw data and statistical analysis code are available in the Supplementary Files.
